# Novel Re(I) tricarbonyl coordination compounds based on 2-pyridyl-1,2,3-triazole derivatives bearing a 4-amino-substituted benzenesulfonamide arm: synthesis, crystal structure, computational studies and inhibitory activity against carbonic anhydrase I, II, and IX isoforms†

**DOI:** 10.1080/14756366.2019.1585835

**Published:** 2019-03-07

**Authors:** Yassine Aimene, Romain Eychenne, Sonia Mallet-Ladeira, Nathalie Saffon, Jean-Yves Winum, Alessio Nocentini, Claudiu T. Supuran, Eric Benoist, Achour Seridi

**Affiliations:** a Laboratoire de Chimie, physique Université du 8 Mai 1945, Guelma, Algérie;; b CNRS, Laboratoire de Synthèse et Physico-Chimie de Molécules d'Intérêt Biologique, SPCMIB, Toulouse, France;; c Université de Toulouse, UPS, Laboratoire de Synthèse et Physico-Chimie de Molécules d'Intérêt Biologique, SPCMIB, Toulouse, France;; d Institut de Chimie de Toulouse (FR 2599), Toulouse, France;; e Institut des Biomolécules Max Mousseron, ENSCM, Université de Montpellier, Montpellier, France;; f Neurofarba Department, Section of Pharmaceutical and Nutriceutical Sciences, Università degli Studi di Firenze, Florence, Italy

**Keywords:** Click chemistry, rhenium(I) complexes, crystal structures, DFT calculations, carbonic anhydrase inhibitors

## Abstract

In this work, two bidentate 2-pyridyl-1,2,3-triazole ligands (**3a** and **3b**) containing a 4-substituted benzenesulfonamide pharmacophore prepared by classical click chemistry procedures, as well as their corresponding rhenium complexes, **4a** and **4b** of general formula [ReCl(CO)_3_(**L**)] (**L **=** 3a** or **3b**) were prepared and fully characterised by spectroscopic methods (IR, NMR, MS, UV-Vis), elemental analysis, X-ray diffraction, and theoretical studies using DFT and TD-DFT methods. In particular, we showed that, in the solid state, the pyridine and the triazole rings of **3b** adopted an uncommon *cis* configuration which stems from intermolecular hydrogen bonds. Preliminary assays demonstrated a promising nanomolar inhibitory activity against carbonic anhydrase isoform IX for both ligands and complexes with a strong affinity *K_i_* of 2.8 nM for ligand **3a**. More interestingly, complex **4b** exhibited a pronounced selectivity against hCA IX over the off-targets hCA I and hCA II which makes this compound a promising potential anticancer drug candidate.

## 1. Introduction

Over recent decades, rhenium(I) tricarbonyl complexes have been intensively studied by the inorganic chemist community, due to their significant photophysical and photochemical properties. These features make them interesting tools for numerous potential practical applications, such as photo sensitisers in solar cells^1^, CO_2_ reduction catalysts^2^, organic light-emitting devices (OLEDs)^3^, luminescence sensors^4^, and CO-releasing moieties (CORMs)^5^. Additionally, radioactive *fac*-[^188^Re(CO)_3_]^+^ complexes have recently drawn the attention of several research groups for their use as therapeutic radiopharmaceuticals^6^.

In this context, the efficient design and synthesis of chelating ligands represent the pivotal key to the successful application of this type of complexes. Most of the reported ligands which coordinate efficiently to the rhenium(I) tricarbonyl core are based on bidentate and tridentate chelating systems including *N*-heteroaromatic nitrogen, oxygen and to a lesser extend sulfur or phosphorus donor atoms^7^. While tridentate ligands lead to the most stable rhenium(I) tricarbonyl complexes, bidentate ligands based on α,α'-diimines are currently very popular. Indeed, their corresponding rhenium complexes exhibit interesting photo-physical properties which can be tuned by small changes in the ligand scaffold or around the rhenium centre by substituting the chlorine bound to the metal by a ternary ligand (generally, cyanides or *N*-heterocycles)^8^. Among efficient bidentate α,α'-diimine chelators, 2,2'-bipyridine (bpy) has been originally used^9^. More recently, similar chelating systems based on pyridine-triazole (pyta and tapy systems^1^) were developed as alternative ligands to 2,2′-bipyridines^10^. Obata et al. first reported that pyta moieties acted like a bipyridine mimic with strongly electron-donating substituents, the corresponding complexes of general formula *fac*-[Re(pyta)(CO)_3_Cl] exhibiting luminescence properties^11^.

The growing interest on pyta (and tapy) architectures is mainly due to their easier synthetic access and functionalization. Unlike bpy systems, it is possible by using a classical copper-catalysed alkyne-azide cycloaddition (CuAAC reaction) not only to generate a large number of this important class of heterocyclic compounds but also to allow different functionalizations of the triazole ring^12^. As an example, we previously reported the preparation of two [M(CO)_3_]^+^ complexes (M = Re and ^99m^Tc) from a pyridyltriazole-based ligand bearing the bioactive (2-methoxyphenyl) piperazine pharmacophore, which is a central nervous system (CNS) receptors-targeted vector. Thus, we demonstrated that (i) both complexes were iso-structural, as expected, (ii) the rhenium complex exhibited fluorescence at room temperature and the radioactive ^99m^Tc-complex presented a suitable lipophilic character for its use as CNS imaging agent^12a^. Given these first results, we decided to explore similar systems as potential human carbonic anhydrase inhibitors (CAIs) by adding a heterocyclic sulfonamide moiety to a pyta scaffold.

While sulfonamides (and bioisosteres sulfamates and sulfamides) are known to possess very efficient carbonic anhydrase inhibitory properties^13^, only a few examples of benzenesulfonamide-based compounds incorporating a [M(CO)_3_]^+^ complex (M = Re and ^99m^Tc) have been reported^14^. Among these examples, Alberto and coworkers showed that simple piano-stool-type rhenium complexes with a pendent arylsulfonamide arm inhibited hCA IX and hCA XII with nanomolar affinities^14c^. Using a similar strategy, i.e. coupling a simple and compact rhenium complex to a 4-substituted benzenesulfonamide moiety, we report here the synthesis of two tricarbonyl rhenium(I) conjugates based on a pyta ligand as well as their full characterisation by spectroscopic methods, X-ray crystallography study and DFT calculations. Preliminary biological assays against cytosolic/membrane-associated carbonic anhydrase isoforms I, II and IX (hCAs I, II and IX) were performed with both ligands and complexes and first results are promising.

## 2. Experimental section

### 2.1. Materials and equipment

All purchased chemicals were of the highest purity commercially available and used without further purification. Analytical grade solvents were used and were not further purified unless specified. Starting materials **1a** and **1b** were purchased from Aldrich Chem. Co. [Re(CO)_5_Cl] was purchased from Acros Organics.

^1^H and ^13 ^C nuclear magnetic resonance (NMR) spectra were recorded with a Bruker Avance 300 (75.5) MHz; chemical shifts are reported in parts per million (ppm) relative to a residual solvent peak and coupling constants (*J*) are given in Hertz (Hz). Multiplicities were recorded as s (singlet), br s (broad singlet), d (doublet), t (triplet), q (quadruplet) … and m (multiplet). Infra-red (IR) spectra were recorded with Perkin–Elmer FTIR 1725 spectrophotometer in the range 4000–400 cm^−1^. Electrospray (ESI) mass spectra were obtained on a Q TRAP Applied Biosystems spectrometer and High-Resolution Mass Spectra (HRMS) on an LCT Premier Waters spectrometer. Microanalysis was performed by the microanalytical department of the Laboratoire de Chimie de Coordination de Toulouse (LCC, Toulouse, France). Electronic spectrum was measured on a Hewlett Packard 8453 temperature-controlled spectrometer in the range 1000–200 nm in methanol solution. Melting points were measured in capillaries using Mettler Toledo and are reported as uncorrected.

Literature methods were used to prepare intermediates **2a** and **2b** ^15^ (if azido compounds are potentially explosive intermediates, in our hands, we never observed hazardous reactions for both azido intermediates).

### 2.2. Syntheses

#### 2.2.1. 4-Azidobenzenesulfonamide (2a).

Yield: 267 mg (93%). ^1^H NMR (300 MHz, DMSO-d_6_): *δ*/ppm = 7.29 (d, *J* = 8.7 Hz, 2H, H_Ar_), 7.36 (s, 2H, NH_2_), 7.82 (d, *J* = 8.6 Hz, 2H, H_Ar_). Spectroscopic analysis is in agreement with reported data^15^.

#### 2.2.2. 4-(Azidomethyl)-benzenesulfonamide (2b).

Yield: 391 mg (85%). ^1^H NMR (300 MHz, DMSO-d_6_): *δ*/ppm = 4.56 (s, 2H, NCH_2_), 7.37 (s, 2H, NH_2_), 7.55 (d, *J* = 8.7 Hz, 2H, H_Ar_), 7.83 (d, *J* = 8.6 Hz, 2H, H_Ar_). Spectroscopic analysis is in agreement with reported data^15^.

**General procedure for CuAAC reaction**: Azide compound (**2a** or **2b**, 1.1 equiv.) and 2-ethynylpyridine (1 equiv.) were suspended in acetonitrile (10 ml). Copper(II) acetate monohydrate (0.2 equiv) and sodium ascorbate (0.4 equiv) were added and the mixture is stirred in the dark at 45 °C overnight (16–18 h). The solvent was then removed under reduced pressure and remaining residues purified by column chromatography on silica gel using CH_2_Cl_2_/MeOH (95:5) as eluent.

#### 2.2.3. 4-(4–(2-pyridyl)-1*H*-1,2,3-triazol-1-yl)-benzenesulfonamide (3a)

Two fifty milligams (1.26 mmol) of **2a** and 116 μL (1.15 mmol) of 2-ethynylpyridine with 46 mg (0.23 mmol) of Cu(OAc)_2_.H_2_O and 91.4 mg (0.46 mmol) of sodium ascorbate yielded the desired compound **3a** as a yellow solid. Suitable crystals of **3a** for X-ray crystal structure determination were grown by slow evaporation of methanol solution.

Yield: 236 mg (69%). mp 244–248 °C. ^1^H NMR (300 MHz, DMSO-d_6_): *δ*/ppm = 7.43 (ddd, *J* = 7.6, 4.8, 1.2 Hz, 1H, H_pyr_), 7.54 (s, 2H, NH_2_), 7.97 (td, *J* = 7.7, 1.8 Hz, 1H, H_pyr_), 8.04 (d, *J* = 8.7 Hz, 2H, H_Ar_), 8.14 (dt, *J* = 7.9, 1.1 Hz, 1H, H_pyr_), 8.26 (d, *J* = 8.7 Hz, 2H, H_Ar_), 8.63-8.71 (m, 1H, H_pyr_), 9.46 (s, 1H, H_ta_). ^13 ^C NMR (75 MHz, DMSO-d_6_): *δ*/ppm = 119.9, 123.5, 137.4, 149.2 (CH_pyr_), 120.5, 127.5 (CH_Ar_), 121.6 (CH_ta_) 138.6, 144.0 (C_Ar_), 148.5 (C_pyr_), 149.8 (C_ta_). ESI^+^-MS: *m/z* = 302.1 [M + H]^+^, 324.1 [M + Na]^+^. HRMS calculated for C_13_H_12_N_5_O_2_S ([M + H]^+^) 302.0712; found 302.0716. Elemental analysis for C_13_H_11_N_5_O_2_S: calculated (found) C, 51.82 (51.64); H, 3.68 (3.63); N, 23.24 (22.26).

#### 2.2.4. 4–(4-(2-pyridyl)-1*H*-1,2,3-triazol-1-ylmethyl)-benzenesulfonamide (3b)

Two twenty milligrams (1.04 mmol) of **2b** and 95 μL (0.94 mmol) of 2-ethynylpyridine with 38 mg (0.19 mmol) of Cu(OAc)_2_.H_2_O and 75.5 mg (0.38 mmol) of sodium ascorbate yielded the desired compound **3b** as a yellow solid. Suitable crystals of **3b** for X-ray crystal structure determination were grown by slow evaporation of methanol solution.

Yield: 180 mg (61%). mp 192–195 °C. ^1^H NMR (300 MHz, DMSO*-*d_6_): *δ*/ppm = 5.78 (br s, 2H, NCH_2_), 7.29–7.41 (m, 3H, H_pyr_, NH_2_), 7.53 (d, *J* = 8.7 Hz, 2H, H_Ar_), 7.83 (d, *J* = 8.7 Hz, 2H, H_Ar_),7.90 (ddd, J = 7.9, 7.5, 1.8 Hz, 1H, H_pyr_), 8.04 (dt, *J* = 7.9, 1.1 Hz, 1H, H_pyr_), 8.60 (ddd, *J* = 4.8, 1.8, 1.0 Hz, 1H, H_pyr_), 8.73 (s,1H, H_ta_). ^13 ^C NMR (75 MHz, DMSO*-*d_6_): *δ*/ppm = 52.4 (CH_2_), 119.4, 123.7, 137.2, 149.6 (CH_pyr_), 123.1 (CH_ta_), 126.2, 128.4 (CH_Ar_), 139.7, 143.9 (C_Ar_), 147.6 (C_pyr_), 149.8 (C_ta_). ESI^+^-MS: *m/z* = 316.1 [M + H]^+^. HRMS calculated for C_14_H_14_N_5_O_2_S ([M + H]^+^) 316.0868, found 316.0868. Elemental analysis for C_14_H_13_N_5_O_2_S: calculated (found) C, 53.32 (53.19); H, 4.16 (3.94); N, 22.21 (21.49).

**General procedure for complexation reaction**: A solution of **3a**/**3b** (1 equiv) and commercial [Re(CO)_5_Cl] (1.1 equiv.) in methanol (8 ml) was stirred 12 h at 65 °C. After cooling to room temperature, the solution was concentrated until 3 ml and then a precipitate was obtained. Five millilitres of methanol was added to the precipitate. The mixture was heated, then cooled and stored at 4 °C for 3 h and then a supernatant was carefully removed. This process was repeated three times before the precipitate was dried under vacuum.

#### 2.2.5. [(3a)Re(CO)_3_Cl], (4a)

**3a** (70 mg, 0.23 mmol) and [Re(CO)_5_Cl] (92.5 mg, 0.25 mmol) yielded the desired complex **4a** as a white solid. Suitable crystals of **4a** for X-ray crystal structure determination were grown by slow evaporation of methanol solution.

Yield: 95 mg (67%). mp >300 °C. ^1^H NMR (300 MHz, DMSO*-*d_6_): *δ*/ppm = 7.64 (s, 2H, NH_2_), 7.71 (ddd, *J* = 7.3, 5.5, 1.6, 1H, H_pyr_), 8.14 (d, *J* = 8.8 Hz, 2H, H_Ar_), 8.25 (d, *J* = 8.8 Hz, 2H, H_Ar_), 8.31 (ddd, *J* = 7.9, 1.6, 0.8 Hz, 1H, H_pyr_), 8.38 (td, *J* = 7.7, 1.5 Hz, 1H, H_pyr_), 9.04 (dd, *J* = 5.6, 1.2 Hz, 1H, H_pyr_), 10.08 (s, 1H, H_ta_). ^13 ^C NMR (75 MHz, DMSO*-*d_6_): *δ*/ppm = 121.4, 127.8 (CH_Ar_), 122.7, 126.9, 137.4, 149.2 (CH_pyr_), 124.6 (CH_ta_), 141.1, 145.5 (C_Ar_), 148.3 (C_pyr_), 153.3 (C_ta_), 189.4, 196.5, 197.4 (CO). IR: ν_CO_=2027, 1931, 1905 cm^−1^, ESI^+^-MS: *m/z* = 570.0 [M-Cl]^+^, 627.9 [M + Na]^+^. HRMS calculated for C_16_H_11_N_5_O_5_SClRe ([M + Na]^+^) 627.9597, found 627.9616. Elemental analysis for C_16_H_11_N_5_O_5_SClRe: calculated (found) C, 31.66 (31.37); H, 1.83 (1.44); N, 11.54 (11.10).

#### 2.2.6. [(3b)Re(CO)_3_Cl], (4b)

**3b** (75 mg, 0.238 mmol) and [Re(CO)_5_Cl] (95 mg, 0.26 mmol) yielded the desired complex **4b** as a white solid. Suitable crystals of **4b** for X-ray crystal structure determination were grown by slow evaporation of methanol solution.

Yield: 98 mg (66%). mp 268–271 °C. ^1^H NMR (300 MHz, DMSO-d_6_): *δ*/ppm = 6.00 (s, 2H, NCH_2_), 7.41 (s, 2H, NH_2_), 7.60–7.68 (m, 3H, 1H_pyr_, 2H_Ar_), 7.90 (d, *J* = 8.3 Hz, 2H, H_Ar_), 8.19–8.37 (m, 2H, 2H_pyr)_, 8.96 (dd *J* = 5.4, 1.3 Hz, 1H, H_pyr_), 9.31 (s, 1H, H_ta_). ^13 ^C NMR (75 MHz, DMSO-d_6_): *δ*/ppm = 54.1 (CH_2_), 122.8, 126.6, 137.8, 148.6 (CH_pyr_), 126.2 (CH_ta_), 126.4, 129.2 (CH_Ar_), 140.7, 144.5 (C_Ar_), 148.6 (C_pyr_), 153.0 (C_ta_), 189.5, 196.7, 197.5 (CO). IR (KBr): ν_CO_=2029, 1920, 1902 cm^−1^. ESI^+^-MS: *m/z* = 584.0 [M-Cl]^+^, 642 [M + Na]^+^. HRMS calculated for C_17_H_13_N_5_O_5_NaSClRe ([M + Na]^+^) 641.9753, found 641.9750. Elemental analysis for C_17_H_13_N_5_O_5_SClRe: calculated (found) C, 32.88 (32.79); H, 2.11 (1.84); N, 11.28 (10.97).

### 2.3. Crystal structure determination

X-ray intensity data of ligands **3a**, **3b** and corresponding rhenium complexes **4a**, **4b** were collected on a Bruker D8 VENTURE diffractometer (MA, USA) and using graphite monochromated Mo Kα radiation (*λ* = 0.71073 Å) for **3b**, **4a** and **4b** and Cu Kα radiation (*λ* = 1.54178 Å) for **3a** at 193 K. The semi-empirical absorption corrections were employed [SADABS, Programme for data correction, Bruker-AXS]. Crystallographic data and refinement details are given in Table 1. The structures were solved using an intrinsic phasing method^16^, and refined by full matrix least squares procedures on F^2^. All non-hydrogen atoms were refined with anisotropic displacement coefficients. The hydrogen atoms bound to carbon atoms were placed in calculated positions and treated as riding on their parent atoms with d(C–H)=0.93 Å, U_iso_(H)=1.2 U_eq_(C) (aromatic); and d(C–H)=0.96 Å, U_iso_(H)=1.5 U_eq_(C) (methyl). The methyl groups were allowed to rotate about their local threefold axis. The ShelX software package^16^ was used for the calculations.

**Table 1.** Crystal data and structure refinement for **3a**, **3b**, **4a,** and **4b**.

	Pyta ligands		Rhenium complexes	
	**3a**	**3b**	**4a**	**4b**
Empirical formula	C_13_H_11_N_5_O_2_S	C_14_H_13_N_5_O_2_S	C_16_H_11_ClN_5_O_5_ReS	C_17_H_13_ClN_5_O_5_ReS, CH_4_O
Formula weight	301.33	315.35	607.01	653.08
T [K]	193(2)	193(2)	193(2)	193(2)
Wavelength [Å]	1.54178	0.71073	0.71073	0.71073
Crystal system	Triclinic	Monoclinic	Orthorhombic	Triclinic
Space group	*P*$\bar{1}$	*P*2_1_/c	*P*bcn	*P*$\bar{1}$
Unit cell dimensions [Å, °]	a = 6.3545(2)	a = 15.8111(9)	a = 19.1728(15)	a = 7.7195(5)
	b = 7.0441(2)	b = 10.6289(5)	b = 15.1735(11)	b = 12.1870(7)
	c = 29.2801(7)	c = 8.4974(4)	c = 13.1204(11)	c = 13.1222(8)
	α = 95.856(2)	α = 90	α = 90	α = 108.398(2)
	β = 90.497(2)	β = 104.485(2)	β = 90	β = 98.897(2)
	γ = 96.930(2)	γ = 90	γ = 90	γ = 103.123(2)
V [Å^3^]	1293.97(6)	1382.64(12)	3817.0(5)	1106.07(12)
Z	4	4	8	2
*ρ_calcd_* [Mg/m^3^]	1.547	1.515	2.113	1.961
μ [mm^−1^]	2.355	0.250	6.657	5.754
Max. and min. transm.	0.7523 and 0.6483	0.7461 and 0.7194	0.7461 and 0.5932	0.7461 and 0.4955
F(000)	624	656	2320	632
Crystal size [mm]	0.28 × 0.04 × 0.02	0.22 × 0.12 × 0.08	0.20 × 0.06 × 0.02	0.30 × 0.28 × 0.06
θ range [°]	3.035–63.142	3.131–30.590	3.102–30.581	3.314–30.535
Limiting indices	−7 ≤ h ≤ 7	−21 ≤ h ≤ 22	−27 ≤ h ≤ 27	−11 ≤ h ≤ 11
	−8 ≤ k ≤ 8	−15 ≤ k ≤ 15	−20 ≤ k ≤ 21	−16 ≤ k ≤ 17
	−33 ≤ l ≤ 33	−12 ≤ l ≤ 12	−18 ≤ l ≤ 18	−18 ≤ l ≤ 17
Reflections collected	12039	21683	116299	36946
Unique reflections (R_int_)	4055 [0.0508]	4237 [0.0442]	5852 [0.1036]	6724 [0.0198]
Completeness to 2θ = 63.142° for **3a**	96.3 %	99.8 %	99.8 %.	99.3 %
=25.242° for **3b**				
=25.242° for **4a**				
=25.242°for **4b**				
Data / restraints / parameters	4055 / 6 / 395	4237 / 0 / 207	5852 / 0 / 270	6724 / 0 / 299
Goodness-of-fit (GOF) on F^2^	1.048	0.859	1.005	1.138
Final *R* indices [*I > 2σ(I)*]	R_1_=0.0478	R_1_=0.0443	R_1_=0.0365	R_1_=0.0196
	wR_2_=0.0932	wR_2_=0.1223	wR_2_=0.0531	wR_2_=0.0464
*R* indices (all data)	R_1_=0.0782	R_1_=0.0697	R_1_=0.0704	R_1_=0.0218
	wR_2_=0.1049	wR_2_=0.1417	wR_2_=0.0614	wR_2_=0.0474
Largest difference in peak and hole [e Å^−3^]	0.315 and −0.411	0.416 and −0.417	1.279 and −1.402	2.564 and −1.526

**Table 2.** Inhibition data of human CA I, II and IX isoforms with compounds **3a**, **3b**, **4a**, and **4b** in comparison with the standard sulfonamide inhibitor acetazolamide (**AAZ**) by a stopped-flow CO_2_ hydrase assay.

	K*_I_*(nM)^a^			Selectivity ratio	
Compound	hCA I	hCA II	hCA IX	I/IX	II/IX
**3a**	181.3	4.1	2.8	64.75	1.46
**3b**	305.2	56.1	22.5	13.56	2.49
**4a**	3220.5	194.9	18.7	172.2	10.4
**4b**	7146.0	836.5	27.3	261.75	30.64
**AAZ**	*250*	*12*	*25*	*10*	*0.48*

a mean from 3 different assays, by a stopped-flow technique (errors in the range of ±5–10% of the reported value).

Italic values are the references values of AAZ (acetalozamide). These are used to compare with our results.

CCDC 1871452 (**3a**), CCDC 1871453 (**3b**), CCDC 1871454 (**4a**) and CCDC 1871455 (**4b**) contain the supplementary crystallographic data. These data can be obtained free of charge from http://www.ccdc.cam.ac.uk/conts/retrieving.html, or from the Cambridge Crystallographic Data Centre, 12 Union Road, Cambridge CB2 1EZ, UK; tel: +44 (0)1223 336408; fax: +44 (0)1223 336033; or e-mail: deposit@ccdc.cam.ac.uk.

### 2.4. Computational details

The reported calculations for the studied tricarbonyl rhenium(I) complexes **4a** and **4b** were carried out using the GAUSSIAN 09 programme package^17^ with the aid of the GaussView visualisation program^18^. Full geometries optimizations were performed in gas-phase and methanol solvent without any symmetry restrictions with the DFT method using the hybrid B3LYP (Becke, three-parameter, Lee-Yang-Parr) functional^19^. The pseudo-potential (ECP) LANL2DZ^20^ was employed to describe the electrons of Re atom while the other atoms (H, C, O, N and S) were assigned by the standard 6–31 G (d) basis set. The optimised geometries were evaluated using vibrational frequencies calculations to ensure that the true local minimum was attained and all eigenvalues are positive. Natural bond orbital analysis was carried out using NBO code included in GAUSSIAN 09^21^. At the optimised structures, time-dependent density functional theory (TD-DFT) method^22^ using B3LYP functional was applied to calculate the vertical excitation energies and corresponding oscillator strengths. In addition, the solvent effect (methanol) was modelled using the polarisable continuum model with the integral equation formalism (IEF–PCM)^23^. The initial geometries were taken from X-ray structures, and all calculations were based on the optimised geometries.

### 2.5. Carbonic anhydrase inhibition assays

An Applied Photophysics stopped-flow instrument was used for assaying the CA catalysed CO_2_ hydration activity. Phenol red (at a concentration of 0.2 mM) was used as indicator, working at the absorbance maximum of 557 nm, with 20 mM Hepes (pH 7.5) as buffer, and 20 mM Na_2_SO_4_ (for maintaining the constant ionic strength), following the initial rates of the CA-catalysed CO_2_ hydration reaction for a period of 10–100 s. The CO_2_ concentrations ranged from 1.7–17 mM for the determination of the kinetic parameters and inhibition constants. In particular, CO_2_ was bubbled in distilled deionised water for 30 min so that the water was saturated (the concentration at a specific temperature is known from literature). In addition, a CO_2_ assay kit (from Sigma) was used to measure the concentration in variously diluted solutions obtained from the saturated one (which was kept at the same temperature and a constant bubbling during the experiments). For each inhibitor at least six traces of the initial 5–10% of the reaction was used for determining the initial velocity^24^. The uncatalysed rates were determined in the same manner and subtracted from the total observed rates. Stock solutions of inhibitor (0.1 mM) were prepared in distilled-deionised water and dilutions up to 0.01 nM were done thereafter with distilled-deionised water. Inhibitor and enzyme solutions were pre-incubated together for 15 min–2 h (or longer, i.e. 4–6 h) at room temperature (at 4 °C for the incubation periods longer than 15 min) prior to assay, in order to allow for the formation of the E-I complex. The inhibition constants were obtained by non-linear least-squares methods using PRISM 3 and the Cheng-Prusoff equation^25^ ^,^ ^26^, as reported earlier, and represent the mean from at least three different determinations. hCA I was purchased by Sigma-Aldrich and used without further purification, whereas all the other hCA isoforms were recombinant ones obtained in-house as reported earlier^27^ ^,^ ^28^.

## 3. Results and discussion

### 3.1. Synthesis and structural characterisation

**Scheme 1.**
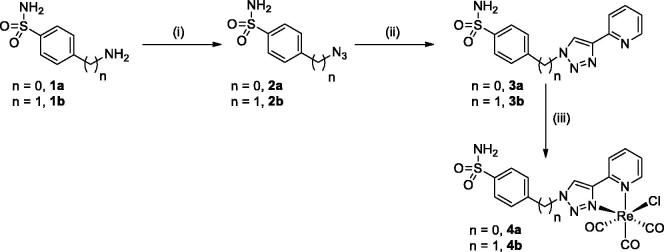
Synthesis of ligands **3a**/**3b** and their corresponding benzenesulfonamide rhenium tricarbonyl complexes **4a**/**4b**. Conditions and reagents: (i) **1a**, NaNO_2_, NaN_3_, HCl/THF/DMF (v/v/v: 1:1:1), 0–25 °C, 1 night (93%) or **1b**, NaN_3_, Tf_2_O, CuSO_4_.5H_2_O, K_2_CO_3_, CH_2_Cl_2_, 0–25 °C, 1 night (85%); (ii) Cu(OAc)_2_.H_2_O, sodium ascorbate, CH_3_CN, 45 °C, 1 night, (**3a**: 69%, 3b: 61%); (iii) [Re(CO)_3_Cl], 65 °C, MeOH, 12h, (**4a**: 67%, 4b: 66%).

The synthesis of rhenium tricarbonyl complexes **4a** and **4b** were readily synthesised following a three-step procedure as depicted in Scheme 1. The ligands **3a** and **3b** were prepared by classical copper-catalysed Huisgen cycloaddition (CuAAC reaction) using the corresponding azido intermediates and commercial 2-ethynylpyridine derivatives. Both azido compounds **2a** and **2b** were obtained in good yields (>80%) by following a procedure reported earlier^15^. By applying classical CuAAC conditions we previously reported for pyta ligands preparation^10b^ (i.e. catalytic system: Cu(OAc)_2_.H_2_O/sodium ascorbate in *tert*-butanol/water mixture or in acetonitrile at room temperature), **3a** and **3b** were obtained in low yield or at trace level. In turn, the yield of the cycloaddition was noticeably improved by performing the reaction at 45 °C in acetonitrile (average yield of 65%). Pyta-based rhenium complexes **4a** and **4b** were finally obtained in modest yields (*c.a.* 66%) by the reaction of commercial rhenium pentacarbonyl chloride with chelating ligands in refluxing methanol during 12 h. All our compounds are stable in the solid state as well as in organic solvents and their stability is not affected by the presence of air or moisture. They were fully characterised by NMR, IR, MS and elemental analysis and exhibited classical spectroscopic features for pyta-based compounds (see experimental section and ESI part, Tables S1–2 for NMR data). Briefly, upon complexation with the tricarbonyl rhenium core, a significant down-field shift (∼0.6 ppm) of the singlet due to the triazole proton and very minor shifts of the hydrogens of the phenyl ring compared to those of the free ligand were observed in proton NMR for each complex, confirming the coordination of the rhenium by the triazolyl unit and the pendent nature of the 4-substituted arylsulfonamide moiety. The pattern of the CO stretching frequencies in the IR spectra (three intense bands in the 2030–1900 cm^−1^ region) confirms the facial arrangement of the CO ligands in the complexes. More interestingly, as reported recently by Sarkar et al., the average value of the CO stretching frequencies gave information on the overall donor strength of the chelating pyta pincer^29^. In our case, both complexes exhibited an average value of 1950 cm^−1^, this value being consistent with those reported for other pyta ligands^29^ and indicating an overall donor strength similar to bpy ligands. Fortunately, we obtained crystals of ligands **3a**/**3b** and complexes **4a**/**4b** suitable for single crystal X-ray diffraction from the slow evaporation of a concentrated sample in methanol solution. Selected crystallographic parameters are listed in Table 1 and selected bond lengths and angles values are gathered in the ESI part (Tables S3–S5).

Both ligands (**3a** and **3b**) were crystallized in triclinic and monoclinic system and their crystals were solved in space groups *P* $\bar{1}$ and *P2*_1_/c, respectively (see ORTEP diagrams in ESI part, Figure S1). As expected, the structural characteristics of **3a**/**3b** are similar to those reported for related pyta ligands^10^ ^a,b,^ ^11^ ^,^ ^12^ ^,^ ^30^. The azo character of the triazolyl ring is confirmed by the N(2)–N(3) distance of the 1,2,3-triazole unit which is shorter than the N(3)–N(4) and N(2)–C(6) bonds (1.314(3) Å vs. 1.357(3) Å and 1.366(4) Å for **3a** with similar bond length values for both molecules of the asymmetric unit, and 1.313(2) Å vs. 1.343(2) Å and 1.363(2) Å for **3b**.

While **3b** is essentially planar with an angle between the least-square planes of the 2-pyridyl and triazole rings of 8.1(1)°, **3a** displays an exceptional deviation between the pyridyl part and the triazolyl entity. The values of the dihedral angle (33.3(1)° and 27.9(1)° for both molecules of the asymmetric unit) are the largest observed for the pyta scaffold compared to the previously reported values (range from 1.56°–15°)^10^ ^a,^ ^11^ ^,^ ^30^. In turn, one of the two molecules of **3a** exhibits a singular planar geometry with respect to the triazolyl moiety and the phenyl group of the benzenesulfonamide unit (dihedral angle value of 3.2(1)° vs. 30.3(2)° for the second molecule of the asymmetric unit). Generally, in pyta scaffold bearing a phenyl group directly connected to the triazole ring, the dihedral angle between the pyridyl and triazole rings is shorter than that between the triazole and phenyl rings (dihedral angle value: *c.a.* 20–30°)^11^ ^,^ ^30a^. A network of inter-molecular hydrogen interactions (see ESI part, Table S6) and π–π interactions could explain these unexpected structural features for **3a** (see ESI part, Table S7).

**Figure 1.**
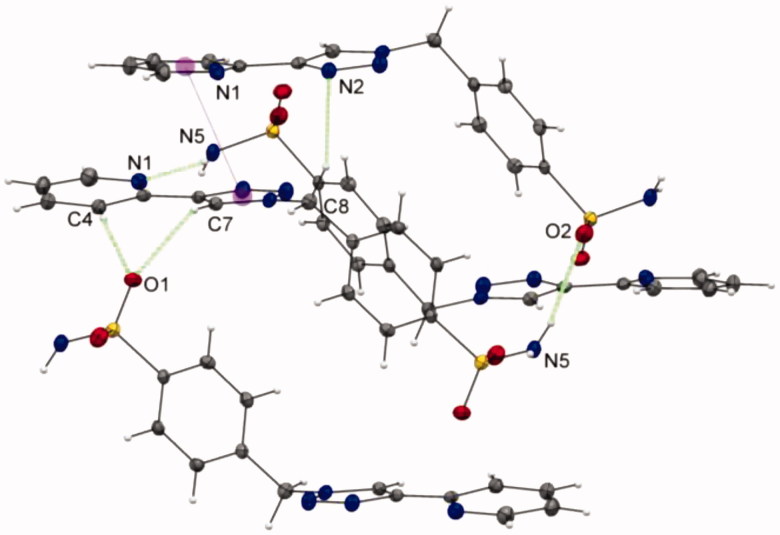
Unprecedented *cis*-configuration of the 2-pyridyl-1,2,3-triazole unit of ligand **3b**; a partial view of the crystal packing of **3b** with N–H•••N and C–H•••O hydrogen bonds and π–π contacts shown as dashed lines. The purple spheres represent the centroids of the rings involved in π–π interactions.

More interestingly, while the nitrogen atoms N(1)/N(2) and N(6)/N(7) of **3a** exhibits a classical anti configuration^11^ ^,^ ^12^ ^,^ ^30^ with minimal electronic repulsions between both nitrogens, an unprecedented *cis* arrangement is clearly adopted by the nitrogen atoms of the pyridine and triazole rings of ligand **3b**, as illustrated in Figure 1. Stabilisation through a network of non-covalent interactions has been invoked to explain thus unexpected arrangement. The single-crystal X-ray structure of **3b** shows two types of hydrogen-bonding interactions (see ESI part, Table S6): stronger intermolecular hydrogen-bonding interactions of the type N–H···N [2.939(2) Å] between the N(5)–H(5b) proton of the sulfonamide moiety and the N(1) atom of the pyridine ring, as well as C–H···O [3.260(2) Å] between the C(7)–H(7) proton of the 1,2,3-triazolyl group and O(1) atom of the sulfonamide moiety. π–π interactions have also been detected in the crystallographic structure for **3b** (see ESI part, Table S7).

**Figure 2.**
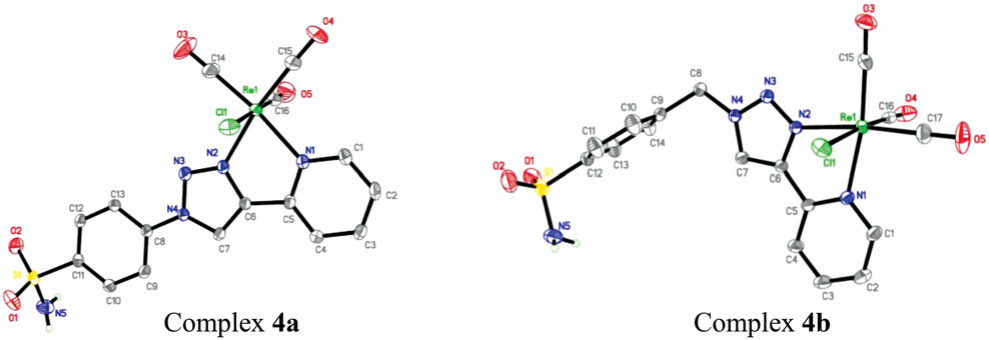
The molecular structures of rhenium complexes **4a** and **4b**. Displacement ellipsoids are drawn at 50% probability, solvent molecule (**4b**) and hydrogen atoms (except H atoms on nitrogen) have been omitted for clarity.

Rhenium complexes **4a** and **4b** shown in Figure 2 (molecular views), crystallise as neutral molecular complexes in the orthorhombic space groups *P*bcn and triclinic space groups *P* $\bar{1},$ respectively. As suggested by the spectroscopic data, each rhenium centre adopts a distorted octahedral coordination geometry. The rhenium atoms are coordinated to two nitrogens of the pyta pincer, three carbonyl donors in a *fac*-orientation and one chlorine atom. The bond lengths and angles for both complexes **4a** and **4b** are unexceptional and similar to those previously reported for other rhenium tricarbonyl complexes based on bidentate ligands^10–12^ ^,^ ^30^. As an example, the average Re-carbonyl bond length is 1.92 Å, and the OC–Re–CO angles range from 87.4(2)° to 90.3(2)° and 88.8(1)° to 90.3(1)° for **4a** and **4b**, respectively. The *trans* bond angles exhibit a moderate distortion from an idealised octahedral geometry (range of 170.3–179.0°), as expected. The Re–Cl bond lengths [2.490(1) Å for **4a**, 2.485(1) Å for **4b**] lie in the upper side of the range (2.454–2.505 Å) found for other rhenium tricarbonyl complexes based on pyta derivatives^10^ ^g,^ ^11^ ^,^ ^12a^. For both complexes **4a** and **4b**, the chelate part is essentially planar with a very slight deviation of 2.5(1)° and 8.2(2)° between the mean planes through the triazolyl and pyridyl rings, respectively. As observed previously, the co-planarity was extended to the benzenesulfonamide pendent arm in **4a**. The 4-substituted aromatic arm shows a dihedral angle of 2.1(1)° with the mean plane of the triazolyl ring, as a result of conjugation^10^ ^b,^ ^31^. In both complexes, a network of π–π interactions and inter-molecular hydrogen bonds between two adjacent complex molecules for **4a** and between two adjacent complex molecules plus the lattice methanol molecule for **4b** are involved in the crystal cohesion (see ESI part, Table S6, and Figure S2).

### 3.2. Computational study

**Figure 3.**
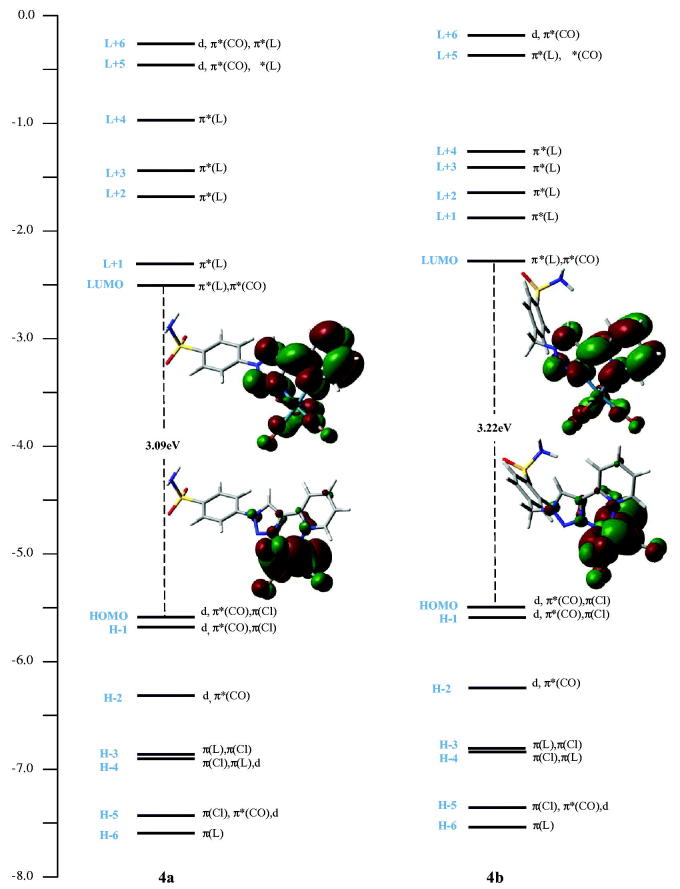
Molecular orbital diagrams of **4a** (Left) and **4b** (Right).

The density functional theory (DFT) and time-dependent density functional theory (TDDFT) calculations were performed in order to assess the impact of the methylene linker (−CH_2_−) between the benzenesulfonamide moiety and the chelate unit of **3b** on the electronic structures of its rhenium complexes (See Tables S8–S10 and Figure S3 in ESI part for more details). While HOMOs energy levels exhibit small changes, the introduction of the methylene spacer in the complex **4b** has an effect on LUMOs energy, as illustrated in Figure 3. Compared to **4a**, the introduction of this methylene linker increased the energy level of LUMO (−2.29 eV) causing wider energy gap (3.22 eV for **4b** *v* *s*. 3.09 eV for **4a**) and a slight blue-shift of the lowest energy absorption band (331 nm for **4b** *vs*. 333 nm for **4a**). The lowest virtual orbitals of complexes are mainly concentrated on the π-anti-bonding orbitals of the chelate and CO ligands. The lowest virtual molecular orbital (LUMO) is centred on π-anti-bonding orbitals of the 2-pyridyl-1,2,3-triazole part and CO ligands. The LUMO + 1 and LUMO + 4 are mainly localised on the full pyta ligand for both complexes. The LUMO + 2 is located on the 2-pyridyl-1,2,3-triazole unit in **4a**, and with a slight contribution from the phenyl ring in the case of **4b**. The LUMO + 3 is wholly centred on phenyl ring for the complex **4a**, whereas for **4b**, it is localised on the full connected ligand.

For **4a** and **4b**, the low-energy absorption bands at 333 and 331 nm have mixed metal-to-ligand [MLCT] and ligand-to-ligand [LLCT] charge transfer character. Accordingly, these electronic transitions originate mainly from the mixed orbitals of the rhenium centre and chlorine to the π-antibonding orbitals of the chelating ligand and carbonyl group, which can be described as {d/π(Cl) → π*(L)/π*(CO) or π*(L)}. The band assignments were presented like those previously reported by other researchers, for related Re(CO)_3_-complexes with similar bidentate ligands^32^.

### 3.3. Carbonic anhydrase inhibitory activity

As mentioned in the introduction, the group of Alberto demonstrated that compact tricarbonyl rhenium complexes acted as excellent carbonic anhydrase IX and XII inhibitors, with nanomolar affinities^14c^. More recently, Tim Storr et al., reported that tricarbonyl rhenium complexes based on a dipyridylamine (DPA) or an iminodiacetate (IDA) ligands with a pendent benzenesulfonamide pharmacophore exhibited interesting CA-IX inhibition (*K_I_* of 37 nM for the best candidate)^14d^.

In our case, both ligands **3a** and **3b** and their corresponding complexes **4a** and **4b** were tested for their efficacy to inhibit physiologically relevant hCA I, II and the tumour-associated hCA IX isoforms, as a preliminary study. The synthesised compounds were screened for their inhibition potential by means of stopped-flow carbon dioxide hydration assay and compared with the clinically used reference drug acetazolamide (AAZ) (Table 2).

Both complexes **4a** and **4b** and the reference drug acetazolamide (AAZ) exhibited similar nanomolar affinities (*ca.* 25 nM) against hCA IX isozyme. Compared to reported affinity values with different tricarbonyl rhenium systems bearing an arylsulfonamide moiety, our values are better than those recently reported by the group of Storr (37–220 nM), in the lower range than those obtained by Babich et al. (3–116 nM) but higher than those obtained by Alberto et al. (3–7 nM)^14b–d^. The introduction of a methylene group between the metallic part and the sulfonamide unit has a slight negative effect on the inhibition values (18.7 nM for **4a** vs. 27.3 nM for **4b**), as previously reported^14c^. Additionally, a better inhibition is observed with ligands **3a** and **3b** compared to their corresponding metallic analogs. In particular, a very strong inhibition is found for ligand **3a**, which displayed 7-fold higher affinity than its rhenium complex **4a** (*K_i_*(**3a**)=2.8 nM vs. *K_i_*(**4a**)=18.7 nM). This unexpected result should be confirmed on other hCA isoforms, the non-metaled free ligands exhibiting generally weaker affinity than that of the corresponding rhenium complexes^14b^. In turn, this first study showed that both rhenium complexes were highly inactive against hCA I and exhibited a low affinity against hCA II but a nanomolar affinity against hCA IX. In terms of selectivity, complex **4b** was our best candidate with promising hCA I/hCA IX and hCA II/hCA IX ratios of 261.75 and 30.64, respectively.

## 4. Conclusion

In short, two new bidentate 2-pyridyl-1,2,3-triazole ligands with a pendent 4-substituted benzenesulfonamide arm and their corresponding Re(CO)_3_-complexes were successfully synthesised. All the prepared compounds were fully characterised by classical spectroscopic methods and X-ray, as well as theoretical studies for both complexes. While in the solid state, rhenium complexes showed classical distorted octahedral geometry, ligand **3b** adopted an uncommon *cis*-configuration arising from a network of intermolecular hydrogen-bonding interactions. DFT calculations revealed that the presence of methylene (−CH_2_−) linker between the benzenesulfonamide moiety and the chelate unit has a slight effect on the absorption bands position of **4b** as well as the HOMO-LUMO gap which is slightly blue-shifted compared to **4a**.

Preliminary investigation on the inhibitory activity of these compounds against the cytosolic human carbonic anhydrase I, II and the membrane-associated isoforms IX (hCAs I, II and IX) revealed an activity in the nanomolar range for hCA IX isozyme. Surprisingly, better inhibition was found with ligands compared to rhenium complexes. In particular, this preliminary study showed that ligand **3a** exhibited a strong affinity *K_i_* of 2.8 nM for hCA IX. Additionally, complex **4b** exhibited a pronounced selectivity against hCA IX over the off-targets isoforms hCA I and hCA II which makes this compound a promising potential anticancer drug candidate. Further *in vitro* studies against other hCA isoforms with both ligands and rhenium complexes and other derivatives based on the same scaffold are under progress in order to rationalise these first biological results.

## Supplementary Material

Supplemental Material

## Note

1 Pyta and tapy mean 4-(2-pyridyl)-1,2,3-triazole and 1-(2-pyridyl)-1,2,3-triazole, respectively.
