# Supplementary material for: Novel Re(I) tricarbonyl coordination compounds based on 2-pyridyl-1,2,3-triazole derivatives bearing a 4-amino-substituted benzenesulfonamide arm: synthesis, crystal structure, computational studies and inhibitory activity against carbonic anhydrase I, II, and IX isoforms†
Source: J Enzyme Inhib Med Chem. 2019 Mar 7;34(1):773–82. doi: 10.1080/14756366.2019.1585835 (PMC6407592; doi:10.1080/14756366.2019.1585835)
Supplement: Supplemental Material [file IENZ_A_1585835_SM4621.pdf]

# Novel Re(I) tricarbonyl coordination compounds based on 2-pyridyl-1,2,3-triazole derivatives bearing a 4-amino-substituted benzenesulfonamide arm: Synthesis, crystal structure, computational studies and inhibitory activity against carbonic anhydrase I, II and IX isoforms.

Yassine Aimene,[a,b,c] Romain Eychenne,[b,c] Sonia Mallet-Ladeira,[d] Nathalie Saffon,[d] Jean-Yves Winum,[e] Alessio Nocentini,[f] Claudiu T. Supuran,[f] Eric Benoist†,[b,c] Achour Seridi[1],[a]

[a.] *Laboratoire de Chimie physique Université du 8 Mai 1945, B.P.401, Guelma, Algérie*

[b] *CNRS, Laboratoire de Synthèse et Physico-Chimie de Molécules d'Intérêt Biologique, SPCMIB, UMR 5068, 118, Route de Narbonne, F-31062 Toulouse Cedex 9, France,*

[c] *Université de Toulouse, UPS, Laboratoire de Synthèse et Physico-Chimie de Molécules d'Intérêt Biologique, SPCMIB, UMR 5068, 118, Route de Narbonne, F-31062 Toulouse Cedex 9, France,*

[d] *Institut de Chimie de Toulouse (FR 2599), 118 Route de Narbonne, Toulouse 31062 Cedex 09, France.*

[e] *Institut des Biomolécules Max Mousseron UMR 5247 CNRS, ENSCM, Université de Montpellier, 240 avenue du professeur Emile JeanBrau 34296 Montpellier cedex 5, France*

[f] *Neurofarba Department, Section of Pharmaceutical and Nutriceutical Sciences, Università degli Studi di Firenze, Via Ugo Schiff 6, 50019 Sesto Fiorentino, Florence, Italy.*

[1] Corresponding author: Tel +213666821635: E-mail addresses : benoist@chimie.ups-tlse.fr (E. Benoist) ID: orcid.org/0000-0003-0764-9024 or seridi_a@yahoo.fr (A. Seridi) ID: orcid.org/0000-0002-2288-544X

**Table S1.** Selected $^{1}H$, $^{13}C$ NMR data in DMSO-$d_6$ (δ in ppm) of the ligands **3a** and **3b** and their corresponding complexes **4a** and **4b**

**Table S2.** Selected experimental and calculated IR data of the carbonyl groups [*ν (C≡O)* ($cm^{-1}$)/KBr] for **4a** and **4b**

**Table S3.** Selected experimental bond lengths [Å] and angles [°] for **3a** and **3b**

**Tables S4** and **S5.** X-ray, calculated bond lengths [Å] and the angles [°] of **4a** and **4b**

**Table S6.** Hydrogen bonds for **3a**, **3b**, **4a** and **4b** [Å and °]

**Table S7.** π-π interactions for **3a**, **3b**, **4a** and **4b** [Å and °]

**Table S8.** Atomic charges [a.u], occupancy and hybridization of the natural bond orbitals between the rhenium and carbonyl ligands for **4a** and **4b**

**Table S9.** The natural populations of the 5*d* orbitals of the Re center in complexes **4a** and **4b**

**Table S10.** Selected wavelengths ($\lambda_{exp}$ and $\lambda_{cal}$), calculated excitation energies (E), oscillator strengths (f), and dominant excitation character for low-lying singlet of **4a** and **4b**

**Figure S1.** Labelled ORTEP diagram of ligand **3a** (top) and **3b** (bottom) with thermal ellipsoids shown at 50% probability and hydrogen omitted for clarity

**Figure S2.** Partial view of the crystal packing of **4a** and **4b** with hydrogen bonds and π–π contacts shown as dashed lines

**Figure S3.** Experimental (top) and electronic transitions calculated with TDDFT/B3LYP method (down) for **4a** and **4b**

**Table S1.** Selected $^{1}H$, $^{13}C$ NMR data in DMSO-$d_6$ (δ in ppm) of the ligands **3a** and **3b** and their corresponding complexes **4a** and **4b**

| | Phenyl moiety | | | | Triazole ring | | Carbonyl groups | | |
|---|---|---|---|---|---|---|---|---|---|
| *Compound* | $CH_{Ar}$ | | $CH_{Ar}$ | | $CH_{ta}$ | $CH_{ta}$ | $C \equiv O$ | | |
| **3a** | 8.04 | 8.26 | 120.5 | 127.5 | 9.46 | 121.6 | | | |
| **3b** | 7.53 | 7.83 | 126.2 | 128.4 | 8.73 | 123.1 | | | |
| **4a** | 8.14 | 8.25 | 121.4 | 127.8 | 10.08 | 124.6 | 189.4 | 196.5 | 197.4 |
| **4b** | 7.68 | 7.90 | 126.4 | 129.2 | 9.31 | 126.2 | 189.5 | 196.7 | 197.5 |

**Table S2.** Selected experimental and calculated IR data of the carbonyl groups [*ν (C≡O)* (cm$^{-1}$)/KBr] for **4a** and **4b**

| **4a** | | | | | **4b** | | | | |
|---|---|---|---|---|---|---|---|---|---|
| Exp | Calc(Gas) | Δ | Calc(MeOH) | Δ | Exp | Calc(Gas) | Δ | Calc(MeOH) | Δ |
| 2027 | 2101 | 74 | 2092 | 65 | 2029 | 2101 | 72 | 2091 | 62 |
| 1931 | 2032 | 101 | 1985 | 54 | 1920 | 2030 | 110 | 1983 | 63 |
| 1905 | 2002 | 97 | 1969 | 64 | 1902 | 2003 | 101 | 1966 | 64 |

For both complexes **4a** and **4b**, the $\nu_{CO}$ stretching bonds in the 2029-1902 cm$^{-1}$ region were lower than the calculated frequencies by 54-65 cm$^{-1}$ in MeOH solvent and 72-110 cm$^{-1}$ in gas phase. It should be noted that we found a similar mean deviation in previous works.[(1)] The values of the experimental frequencies ($\nu_{CO}$) are in good agreement with the mean of theoretical values. In addition, the calculated frequencies are lower than those in the free ligand (free $\nu_{CO}$ = 2143 cm$^{-1}$), which is consistent with the fact that the back-donation to π* empty molecular orbitals in the carbonyl ligand.[(2)]

(1) M. Wolff, L. Munoz, A. François, C. Carayon, A. Seridi, N. Saffon, C. Picard, B. Machura, E. Benoist, *Dalton Trans.* **2013**, *42*, 7019-7031

(2) P. Cantero-López, L. Le Bras, D. Páez-Hernández, R. Arratia-Pérez. *Dalton Trans*. **2015**, *44*, 20004-20010.

**Table S3.** Selected experimental bond lengths [Å] and angles [°] for **3a** and **3b**

| | **3a** | **3b** |
|---|---|---|
| **Bond Lengths** | | |
| N(2)-N(3) | 1.314(3) | 1.313(2) |
| N(3)-N(4) | 1.357(3) | 1.343(2) |
| N(4)-C(7) | 1.349(4) | 1.343(2) |
| N(2)-C(6) | 1.366(4) | 1.363(2) |
| C(6)-C(7) | 1.364(4) | 1.376(2) |
| C(8)-N(4) | 1.429(4) | 1.467(2) |
| C(6)-C(5) | 1.469(4) | 1.467(2) |
| **Bond Angles** | | |
| C(6)-N(2)-N(3) | 109.4(2) | 108.7(1) |
| N(2)-N(3)-N(4) | 106.5(2) | 107.4(1) |
| N(3)-N(4)-C(7) | 110.9(2) | 111.2(1) |
| N(3)-N(4)-C(8) | 120.3(2) | 119.6(1) |
| N(2)-C(6)-C(5) | 122.7(3) | 121.7(1) |
| C(6)-C(5)-N(1) | 115.4(3) | 116.4(1) |
| C(9)-C(8)-N(4) | 119.0(3) | 110.7(1) |
| C(4)-C(5)-C(6) | 121.1(3) | 121.2(1) |

**Tables S4** and **S5.** X-ray, calculated bond lengths [Å] and the angles [°] of **4a** and **4b**

| **4a** | | DFT | | | |
|---|---|---|---|---|---|
| **Bond lengths** | X-ray | Gas phase | Δ | Methanol | Δ |
| Re(1)-Cl(1) | 2.490(1) | 2.521 | -0.031 | 2.560 | -0.070 |
| Re(1)-C(14) | 1.920(4) | 1.930 | -0.010 | 1.925 | -0.005 |
| Re(1)-C(15) | 1.916(4) | 1.927 | -0.011 | 1.927 | -0.011 |
| Re(1)-C(16) | 1.926(5) | 1.920 | +0.006 | 1.912 | +0.014 |
| Re(1)-N(1) | 2.202(3) | 2.241 | -0.039 | 2.244 | -0.042 |
| Re(1)-N(2) | 2.161(3) | 2.176 | -0.015 | 2.176 | -0.015 |
| O(3)-C(14) | 1.140(5) | 1.158 | -0.018 | 1.161 | -0.021 |
| O(4)-C(15) | 1.142(5) | 1.160 | -0.018 | 1.162 | -0.020 |
| O(5)-C(16) | 1.133(6) | 1.165 | -0.032 | 1.165 | -0.032 |
| **Bond angles** | | | | | |
| C(15)-Re(1)-C(14) | 90.3(2) | 90.83 | -0.49 | 90.68 | -0.34 |
| C(14)-Re(1)-C(16) | 87.4(2) | 91.73 | -4.33 | 91.38 | -3.98 |
| C(15)-Re(1)-C(16) | 88.6(2) | 91.83 | -3.23 | 91.32 | -2.72 |
| C(14)-Re(1)-N(2) | 98.8(2) | 97.60 | +1.23 | 97.57 | +1.26 |
| C(15)-Re(1)-N(2) | 170.3(2) | 169.43 | +0.89 | 170.51 | -0.19 |
| C(16)-Re(1)-N(2) | 94.8(2) | 94.26 | +0.54 | 93.12 | +3.68 |
| C(14)-Re(1)-N(1) | 173.1(2) | 170.32 | +2.77 | 170.93 | +2.16 |
| C(15)-Re(1)-N(1) | 96.6(2) | 96.85 | -0.29 | 97.32 | -0.76 |
| C(16)-Re(1)-N(1) | 93.0(2) | 93.91 | -0.88 | 92.67 | +0.36 |
| N(1)-Re(1)-N(2) | 74.3(1) | 74.14 | +0.12 | 74.11 | +0.15 |
| C(14)-Re(1)-Cl(1) | 94.0(2) | 91.46 | +2.55 | 91.32 | +2.69 |
| C(15)-Re(1)-Cl(1) | 92.6(2) | 91.12 | +1.47 | 90.98 | +1.61 |
| C(16)-Re(1)-Cl(1) | 178.1(1) | 175.62 | +2.49 | 176.43 | +1.68 |
| N(1)-Re(1)-Cl(1) | 85.4(1) | 82.53 | +2.88 | 84.33 | +1.08 |
| N(2)-Re(1)-Cl(1) | 83.8(1) | 82.34 | +1.42 | 84.20 | -0.44 |
| O(3)-C(14)-Re(1) | 175.6(5) | 178.15 | -2.55 | 178.28 | -2.68 |
| O(4)-C(15)-Re(1) | 177.6(4) | 177.41 | +0.19 | 178.36 | -0.76 |
| O(5)-C(16)-Re(1) | 176.6(4) | 179.35 | -2.75 | 179.83 | -3.23 |

| **4b** | | DFT | | | |
|---|---|---|---|---|---|
| **Bond lengths** | X-ray | Gas phase | Δ | Methanol | Δ |
| Re(1)-Cl(1) | 2.485(1) | 2.527 | -0.0416 | 2.5624 | -0.077 |
| Re(1)-C(15) | 1.919(2) | 1.930 | -0.011 | 1.924 | -0.005 |
| Re(1)-C(17) | 1.917(3) | 1.927 | -0.010 | 1.926 | -0.009 |
| Re(1)-C(16) | 1.927(3) | 1.919 | +0.008 | 1.912 | +0.015 |
| Re(1)-N(1) | 2.202(2) | 2.242 | -0.039 | 2.244 | -0.042 |
| Re(1)-N(2) | 2.149(2) | 2.178 | -0.029 | 2.177 | -0.028 |
| O(3)-C(15) | 1.154(3) | 1.158 | -0.004 | 1.161 | -0.007 |
| O(5)-C(17) | 1.152(3) | 1.160 | -0.008 | 1.162 | -0.010 |
| O(4)-C(16) | 1.132(3) | 1.165 | -0.033 | 1.165 | -0.033 |
| **Bond angles** | | | | | |
| C(15)-Re(1)-C(17) | 89.3(1) | 90.80 | -1.46 | 90.72 | -1.38 |
| C(15)-Re(1)-C(16) | 90.3(1) | 91.60 | -1.32 | 91.36 | -1.08 |
| C(16)-Re(1)-C(17) | 88.8(1) | 91.78 | -2.94 | 91.29 | -2.45 |
| C(15)-Re(1)-N(2) | 96.9(1) | 97.73 | -0.81 | 97.45 | -0.53 |
| C(17)-Re(1)-N(2) | 173.2(1) | 169.13 | +4.03 | 170.58 | +2.58 |
| C(16)-Re(1)-N(2) | 93.9(1) | 94.69 | -0.83 | 93.17 | +0.69 |
| C(15)-Re(1)-N(1) | 170.9(1) | 170.56 | +0.37 | 170.76 | +0.17 |
| C(17)-Re(1)-N(1) | 99.4(1) | 96.85 | +2.57 | 97.45 | +1.97 |
| C(16)-Re(1)-N(1) | 92.2(1) | 93.66 | -1.44 | 92.73 | -0.51 |
| N(1)-Re(1)-N(2) | 74.2(1) | 74.05 | +0.16 | 74.06 | +0.15 |
| C(15)-Re(1)-Cl(1) | 94.9(1) | 91.47 | +3.43 | 91.46 | +3.44 |
| C(17)-Re(1)-Cl(1) | 92.0(1) | 91.40 | +0.60 | 90.81 | +1.19 |
| C(16)-Re(1)-Cl(1) | 174.7(1) | 175.54 | -0.79 | 176.45 | -1.70 |
| N(1)-Re(1)-Cl(1) | 82.5(1) | 82.85 | -0.31 | 84.15 | -1.61 |
| N(2)-Re(1)-Cl(1) | 84.7(1) | 81.71 | +3.04 | 84.34 | +0.41 |
| O(3)-C(15)-Re(1) | 178.4(2) | 178.16 | +0.24 | 178.22 | +0.18 |
| O(5)-C(17)-Re(1) | 179.0(3) | 177.41 | +1.59 | 178.42 | +0.58 |
| O(4)-C(16)-Re(1) | 177.2(2) | 179.38 | -2.18 | 179.80 | -2.60 |

The predicted

geometrical parameters of **4a** and **4b** in the gas phase and methanol solvent are found and compared with the experimental X-ray single crystal data. We can see that the calculated values which included the solvent effect did not differ statistically from the gas phase results, where the structures still keep similar geometrical features. Incontestably, P3LYB/LANL2DZ method displays a very good estimation of Re–C distances, with some variation ranging from 0.006 to 0.011 Å in vacuum and 0.005 to 0.015Å in methanol medium for the two Re-compounds. Whereas, the variation of Re–N distance, is 0.015[0.015] and 0.039[0.042] Å in the gas phase [MeOH solvent] for **4a** and 0.029[0.028] and 0.039[0.042] Å in the gas phase [MeOH solvent] for **4b**, the Re–Cl distances errors for **4a** and **4b** are 0.031 and 0.0416 Å in the gas phase and 0.070 and 0.077 Å in the methanol solvent, respectively. Turki *et al*. noted that the larger errors recorded for the Re-Cl elongation are attributed to a drawback of the DFT technique especially with regard to the dynamic correlation effects, which is important in metal-based complexes including a polar M-Cl bond.(3)

In our case, the differences between modeled results and experimental data are within the range of allowed errors considering the environmental factors such as solvent impact and crystal packing. Moreover, the values of calculated bond lengths (Re–C, Re–N and Re–Cl) and bond angles for both transition metal complexes **4a** and **4b** were similar, which clearly indicates that methylene ($-CH_2-$) spacer linked to a chelate part does not affect the geometry of structures around the rhenium(I) center. In each complex equatorial Re-C distances are larger than the axial Re-C distances evidenced the back-donation from the rhenium center to π-anti bonding orbital in the carbonyl *trans* to the chloride ion.

(3) M. Turki, C. Daniel, S. Záliš, A. Vlček, J. van Slageren, D.J. Stufkens, *J. Am. Chem. Soc*. **2001**, *123*, 11431-11440.

**Table S6.** Hydrogen bonds for **3a**, **3b**, **4a** and **4b** [Å and °]

| | **D** **A** | **D—H** [Å] | **H•••A** [Å] | **D•••A** [Å] | **D—H•••A** [°] |
|---|---|---|---|---|---|
| **3a** | N(10)-H(10A)...N(2)#1 | 0.90(3) | 2.11(3) | 3.009(4) | 173(3) |
| | N(10)-H(10B)...N(1) | 0.87(4) | 2.11(4) | 2.956(4) | 167(3) |
| | N(5)-H(5B)...N(6) | 0.82(4) | 2.18(4) | 2.971(4) | 162(3) |
| | N(5)-H(5A)...N(7)#1 | 0.89(4) | 2.15(4) | 3.047(4) | 178(3) |
| **3b** | C(4)-H(4)...O(1)#1 | 0.95 | 2.49 | 3.376(2) | 154.9 |
| | C(7)-H(7)...O(1)#1 | 0.95 | 2.50 | 3.260(2) | 137.5 |
| | C(8)-H(8B)...N(2)#2 | 0.99 | 2.53 | 3.413(2) | 149.1 |
| | N(5)-H(5B)...N(1)#3 | 0.89(2) | 2.16(2) | 2.939(2) | 145.7(18) |
| | N(5)-H(5A)...O(2)#2 | 0.83(2) | 2.42(2) | 3.196(2) | 156(2) |
| **4a** | N(5)-H(5a)...N(3)#1 | 0.88(5) | 2.58(5) | 3.267(6) | 135(4) |
| | N(5)-H(5b)...Cl(1)#2 | 0.78(5) | 2.50(6) | 3.271(5) | 169(5) |
| **4b** | N(5)-H(5a)...O(6)#2 | 1.00(4) | 1.90(4) | 2.889(5) | 170(3) |
| | N(5)-H(5b)...Cl(1)#1 | 0.95(5) | 2.64(5) | 3.347(3) | 132(3) |
| | O(6)-H(6a)...Cl(1) | 0.84 | 2.33 | 3.134(3) | 161.0 |

Symmetry codes (**3a**) : #1 x+1, y, z ; (**3b**) : #1 -x+1, -y+1, -z+1 ; #2 x, -y+3/2, z+1/2 ; #3 -x+1, y-1/2, -z+3/2 ; (**4a**) : #1 -x+2, -y+1, -z ; #2 x+1/2, y-1/2, -z+1/2 ; (**4b**) : #1 -x+1, -y, -z+1 ; #2 x+1, y, z.

**Table S7.** π-π interactions for **3a**, **3b**, **4a** and **4b**

*Cg*1, *Cg*2, *Cg*3, *Cg*4 and *Cg*5 are the centroids of the N2-N4/C7/C6, N1/C1-C5, C8-C13, N6/C14-C18 and C21-C26 rings, respectively

| | ***Cg*(I)** | ***Cg*(J)** | **Distance *Cg*(I)•••*Cg*(J)** [Å] | **Interplanar distance** [Å] | **β** [°] |
|---|---|---|---|---|---|
| **3a** | *Cg*(1) | *Cg*(5) | 3.844(1) | 3.463 | 25.7 |
| | *Cg*(2) | *Cg*(2)#1 | 3.761(1) | 3.376 | 26.3 |
| | *Cg*(3) | *Cg*(5)#2 | 3.775(1) | 3.435 | 24.5 |
| | *Cg*(4) | *Cg*(4)#3 | 3.679(1) | 3.410 | 22.1 |
| **3b** | *Cg*(1) | *Cg*(2)#1 | 3.627(1) | 3.419 | 19.5 |
| **4a** | *Cg*(1) | *Cg*(1)#1 | 3.752(2) | 3.364 | 26.3 |
| | *Cg*(2) | *Cg*(2)#1 | 3.676(2) | 3.252 | 27.8 |
| | *Cg*(2) | *Cg*(2)#2 | 3.833(2) | 3.275 | 31.3 |
| | *Cg*(3) | *Cg*(3)#1 | 3.606(2) | 3.270 | 24.9 |
| | *Cg*(3) | *Cg*(3)#3 | 3.964(2) | 3.316 | 33.2 |
| **4b** | *Cg*(1) | *Cg*(1)#1 | 3.758(2) | 3.586 | 17.4 |

β is the angle between the *Cg*(I) → *Cg*(J) vector and the normal to plane I.

Symmetry codes: (**3a**) #1 –x, -y+1, -z+2 ; #2 x, y+1, z ; #3 -x, -y, -z+1 ; (**3b**) #1 x, -y+3/2, z+1/2 ; (**4a**) #1 2-x, y, 1/2-z ; #2 2-x, 2-y, -z ; #3 2-x, 1-y, -z ; (**4b**) #1 2-x, 1-y, 1-z

**Table S8.** Atomic charges [a.u], occupancy and hybridization of the natural bond orbitals between the rhenium and carbonyl ligands for **4a** and **4b**

| Atom | Charge | Bond | Occupancy | Hybridization of NBO |
|---|---|---|---|---|
| **4a** | | | | |
| Re(1) | -0.936 | Re(1)-C(14) | 1.96000(0.13885) | [36.23%]0.6019$(sp^{0.62}\ d^{2.06})_{Re}$+[63.77%]0.7985$(sp^{0.52})_{C}$ |
| C(14) | 0.755 | Re(1)-C(15) | 1.95988(0.14997) | [36.11%]0.6009$(sp^{0.63}\ d^{2.07})_{Re}$+[63.89%] 0.7993 $(sp^{0.52})_{C}$ |
| C(15) | 0.734 | Re(1)-C(16) | 1.91901(0.09509) | [33.90%]0.5822$(sp^{2.84}d^{2.74})_{Re}$+[66.10%] 0.8130 $(sp^{0.52})_{C}$ |
| C(16) | 0.693 | C(14)- O(3) | 1.99595(0.04748) | [30.02%]0.5479$(sp^{2.42})_{C}$+[69.98%] 0.8365$(sp^{1.68})_{O}$ |
| O(3) | -0.445 | | 1.99543(0.18469) | [25.45%]0.5044$(p)_{C}$+[74.55%] 0.8634$(p)_{O}$ |
| O(4) | -0.453 | | 1.99411(0.16298) | [25.89%]0.5088$(p)_{C}$+[74.11%] 0.8609$(p)_{O}$ |
| O(5) | -0.458 | C(15)- O(4) | 1.99642(0.02740) | [30.65%]0.5536$(sp^{2.07})_{C}$+[69.35%]0.8328$(sp^{1.36})_{O}$ |
| N(1) | -0.376 | | 1.99559(0.20746) | [24.82%]0.4982$(p)_{C}$+[75.18%]0.8671$(p)_{O}$ |
| N(2) | -0.169 | | 1.99403(0.18205) | [25.67%]0.5066$(p)_{C}$+[74.33%]0.8622$(p)_{O}$ |
| Cl(1) | -0.412 | C(16)- O(5) | 1.99621(0.03723) | [30.58%]0.5530$(sp^{2.21})_{C}$+[69.42%]0.8332$(sp^{1.47})_{O}$ |
| | | | 1.99640(0.24287) | [24.66%]0.4966$(p)_{C}$ +[75.34%] 0.8680$(p)_{O}$ |
| | | | 1.99419(0.19502) | [25.91%]0.5090$(p)_{C}$+[74.09%] 0.8607$(p)_{O}$ |
| **4b** | | | | |
| Re(1) | -0.935 | Re(1)-C(15) | 1.95997(0.13903) | [36.25%]0.6021$(sp^{0.62}\ d^{2.06})_{Re}$+[63.75%]0.7985$(sp^{0.52})_{C}$ |
| C(15) | 0.755 | Re(1)-C(17) | 1.95981(0.14936) | [36.10%]0.6009$(sp^{0.63}\ d^{2.06})_{Re}$+[63.90%]0.7994$(sp^{0.52})_{C}$ |
| C(17) | 0.733 | Re(1)-C(16) | 1.91915(0.09542) | [33.92%]0.5824$(sp^{2.83}\ d^{2.74})_{Re}$+[66.08%]0.8129$(sp^{0.52})_{C}$ |
| C(16) | 0.692 | C(15)- O(3) | 1.99594(0.04766) | [30.02%]0.5479$(sp^{2.42})_{C}$+[69.98%]0.8366$(sp^{1.68})_{O}$ |
| O(3) | -0.446 | | 1.99544(0.18616) | [25.41%]0.5040$(p)_{C}$+[74.59%]0.8637$(p)_{O}$ |
| O(5) | -0.455 | | 1.99409(0.16305) | [25.89%]0.5088$(p)_{C}$+[74.11%]0.8609$(p)_{O}$ |
| O(4) | -0.471 | C(17)- O(5) | 1.99638(0.02864) | [30.62%]0.5533$(sp^{2.09})_{C}$+[69.38%]0.8330$(sp^{1.37})_{O}$ |
| N(1) | -0.376 | | 1.99565(0.20728) | [24.80%]0.4980$(p)_{C}$+[75.20%]0.8672$(p)_{O}$ |
| N(2) | -0.178 | | 1.99404(0.18404) | [25.66%]0.5065$(p)_{C}$+[74.34%]0.8622$(p)_{O}$ |
| Cl(1) | -0.414 | C(16)- O(4) | 1.99614(0.03898) | [30.53%]0.5526$(sp^{1.49})_{C}$+[69.47%]0.8335$(sp^{2.24})_{O}$ |
| | | | 1.99646(0.24488) | [24.61%]0.4961$(p)_{C}$+[75.39%]0.8683$(p)_{O}$ |
| | | | 1.99418(0.19531) | [25.92%]0.5091$(p)_{C}$+[74.08%]0.8607$(p)_{O}$ |

The calculated charge values on the Re atom (-0.936 a.u. in **4a** and -0.935a.u. in **4b**) are remarkably lower than the formal charge +1, proving a significant charge donation from the ligands. The 5*d* orbitals ($d_{xy}$, $d_{xz}$, $d_{yz}$, $d_{x^2-y^2}$and $d_{z^2}$) of the Re atom give population of 1.38671, 1.54572, 1.54371, 1.15433, 1.04702 and 1.57283, 1.37830, 1.53994, 1.02897, 1.15590 for the formed complexes **4a** and **4b**, respectively (Table S8).

As expected, the carbon atoms of the carbonyl group possess positive charges, while the oxygen atoms are negatively charged. The smallest positive charge is carbon in the *trans* position to the chlorine atom (0.693 for **4a** and 0.692 for **4b**). For each CO group of complexes **4a** and **4b**, one σ and two π natural bond orbitals is characterized for the carbon–oxygen bonds and one orbital for the Rhenium–carbon bonds. The hybridization of NBO analysis shows clearly that Re–C bond orbitals in all cases are polarized towards the carbon atom, and that the detected C–O bond orbitals are strongly polarized towards the oxygen extremity. Each oxygen atom of CO ligand has one lone pair (LP) orbital, and the anti-bonding occupancies are cited in round brackets.

The calculated atomic charges values are consistent with resonance structure $Re^{-}$—$C^{+}$≡O, exhibiting the best bonding interaction between Re atom and carbonyl ligands in **4a** and **4b** complexes.

**Table S9.** The natural populations of the 5d orbitals of the Re center in complexes **4a** and **4b**

| | Occupancy | |
|---|---|---|
| | **4a** | **4b** |
| $d_{xy}$ | 1.38671 | 1.57283 |
| $d_{xz}$ | 1.54572 | 1.37830 |
| $d_{yz}$ | 1.54371 | 1.53994 |
| $d_{x^2-y^2}$ | 1.15433 | 1.02897 |
| $d_{z^2}$ | 1.04702 | 1.15590 |

**Table S10.** Selected wavelengths ($\lambda_{exp}$ and $\lambda_{cal}$), calculated excitation energies (E), oscillator strengths (f), and dominant excitation character for low-lying singlet of **4a** and **4b**

| Excitations | $\lambda_{exp}$[nm] | $\lambda_{calc}$[nm] | $E_{calc}$[eV] | $f_{calc}$ | Character |
|---|---|---|---|---|---|
| **4a** | | | | | |
| H → L | 333 | 379.71 | 3.26 | 0.0066 | d/π(Cl)→π*(L)/π*(CO) [MLCT/LLCT] |
| H-1 → L | | 362.52 | 3.42 | 0.0623 | d/π(Cl)→π*(L)/π*(CO)[MLCT/LLCT] |
| H → L+1 | | 350.48 | 3.54 | 0.0477 | d/π(Cl) → π*(L) [MLCT/LLCT] |
| H-1 → L+1 | | 342.70 | 3.62 | 0.0734 | d/π(Cl) → π*(L) [MLCT/LLCT] |
| H-3 → L | 290 | 277.00 | 4.47 | 0.2856 | π(L)/π(Cl) → π*(L)/π*(CO) [ILCT/LLCT] |
| H-3→L+1 | | 272.05 | 4.56 | 0.3727 | π(L)/π(Cl) → π*(L) [ILCT/LLCT] |
| H-4→L | 246 | 262.14 | 4.73 | 0.2337 | π(Cl)/π(L) → π*(L)/π*(CO) [LLCT/ILCT] |
| H-5→L+1 | | 251.40 | 4.93 | 0.0789 | π(Cl) → π*(L) [LLCT] |
| H-1→L+6 | | | | | d/π(Cl) → d/π*(CO)[LF/MLCT/LLCT] |
| H-6→L | | 246.04 | 5.04 | 0.0916 | π(L)→π*(L)/π*(CO) [ILCT/ LLCT] |
| S28 H-6→L+1 | | 240.19 | 5.16 | 0.0882 | π(L)→π*(L) [ILCT] |
| H-6→L+4 | 206 | 212.90 | 5.82 | 0.0440 | π(L)→π*(L) [ILCT] |
| H-10→L+1 | | 210.85 | 5.88 | 0.0036 | π(L) → π*(L) [ILCT] |
| H-7→L+5 | | 207.83 | 5.96 | 0.0160 | π(L) → d/π*(CO)/π*(L) [LMCT/LLCT] |
| H-13→L+1 | | 207.12 | 5.98 | 0.0159 | π(L) → π*(L) [ILCT] |
| **4b** | | | | | |
| H → L | 331 | 376.27 | 3.29 | 0.0033 | d/π(Cl)→π*(L)/π*(CO) [MLCT/LLCT] |
| H-1 → L | | 359.55 | 3.45 | 0.0737 | d/π(Cl)→π*(L)/π*(CO)[MLCT/LLCT] |
| H-2 → L | | 328.87 | 3.77 | 0.0005 | d →π*(L)/π*(CO)[MLCT] |
| H-3 → L | 274 | 273.65 | 4.53 | 0.0746 | π(L)/π(Cl) → π*(L)/π*(CO)[LLCT/ILCT] |
| H-4 → L | | 271.98 | 4.56 | 0.0916 | π(Cl)/π(L) →π*(L)/π*(CO)[LLCT/ILCT] |
| H-3 → L+3 | | 270.67 | 4.58 | 0.0208 | π(L)/π(Cl) → π*(L) [LLCT/ILCT] |
| H-6 → L | | 264.44 | 4.69 | 0.0482 | π(L) →π*(L)/π*(CO)[ILCT/LLCT] |
| H-7 → L | | 262.37 | 4.72 | 0.0494 | π(L) →π*(L)/π*(CO)[ILCT/LLCT] |
| H-4 → L+5 | 223 | 236.15 | 5.25 | 0.2246 | π(Cl)/π(L) → π*(L)/π*(CO)[LLCT/ILCT] |
| H-11 → L | | 232.68 | 5.33 | 0.3292 | π(L) → π*(L)/π*(CO)[LLCT/ILCT] |
| H-4 → L+1 | | 229.52 | 5.40 | 0.3035 | π(Cl)/π(L) →π*(L) [LLCT/ILCT] |
| H-8 → L | | | | | π(L) → π*(L)/π*(CO)[ILCT/LLCT] |
| H-1 → L+8 | | 222.36 | 5.57 | 0.0946 | d/π(Cl)→π*(L)/π*(CO) [MLCT/LLCT] |
| H-4 → L+2 | | 221.99 | 5.58 | 0.1195 | π(Cl)/π(L) →π*(L) [LLCT/ILCT] |
| H-4 → L+3 | | 216.85 | 5.72 | 0.1005 | π(Cl)/π(L) →π*(L) [LLCT/ILCT] |
| H-7 → L+2 | | 215.33 | 5.76 | 0.1750 | π(L)→π*(L)[ILCT] |
| H-2 → L+8 | 204 | 213.54 | 5.81 | 0.0016 | d/π(Cl)→π*(L)/π*(CO)[MLCT/LLCT] |
| H-6 → L+3 | | 213.05 | 582 | 0.0164 | π(L)→π*(L)[ILCT] |
| H → L+10 | | 211.31 | 5.87 | 0.0068 | d/π(Cl)→π*(L) [MLCT/LLCT] |
| H-7 → L+6 | | 206.55 | 5.99 | 0.0096 | π(L) → d/π*(CO)[LMCT/LLCT] |

For **4a** and **4b**, the low-energy absorption bands at 333 and 331 nm have mixed metal-to-ligand [MLCT] and ligand-to-ligand [LLCT] charge transfer character. Accordingly, these electronic transitions originate mainly from the mixed orbitals of the rhenium center and chlorine to the π-antibonding orbitals of the chelating ligand and carbonyl group, which can be described as {d/π(Cl) → π*(L)/π*(CO) or π*(L)}. The band assignments were presented like those previously reported by other researchers, for related $Re(CO)_3$-complexes with similar bidentate ligands.[(4)]
For complex **4a** the transitions of HOMO-3→LUMO and HOMO-3→LUMO+1, corresponding to intense band at 290 nm, can be described as **{**π(L)/π(Cl) → π*(L)/π*(CO)} and {π(L)/π(Cl) → π*(L)}, respectively, with ligand-ligand/intra-ligands [LLCT/ILCT] charge transfer character. Similarly, the transitions of HOMO-3/HOMO-4/HOMO-6/HOMO-7→LUMO and HOMO-3→LUMO+3, corresponding to the second intense bond at 274 nm for **4b,** possess similar LLCT/ILCT character. It appeared that the experimental absorption to band at 246 and 206 nm for **4a**, 274 and 204 nm for **4b** have been mostly attributed to ligand-to-ligand charge transfer (LLCT), which are occurring from the chelate ligand and chlorine ion to π*(L) or π-antibonding orbital of the CO ligands, metal-to-ligand (MLCT) charge transfer and intra-ligands (IL) excitations. The ligand-ligand/intra-ligands CT [LLCT/IL]

transitions have the major contribution to these bands because of larger values of their oscillator strength "*f*".

(4) (a) M. Obata, A. Kitamura, A. Mori, C. Kameyama, J.A. Czaplewska, R. Tanaka, I. Kinoshita, T. Kusumoto, H. Hashimoto, M. Harada, Y. Mikata, T. Funabiki, S. Yano, *Dalton Trans*., 2008, 3292-3300; (b) P.A. Scattergood, A. Sinopoli, P.I.P. Elliott. *Coord. Chem. Rev*., **2017**, *350*, 136-154.

**Figure S1.** Labelled ORTEP diagram of ligand **3a** (top) and **3b** (bottom) with thermal ellipsoids shown at 50% probability. Hydrogen atoms have been omitted for clarity. Only one of the two independent molecules in the asymmetric unit is shown for clarity (**3a**).

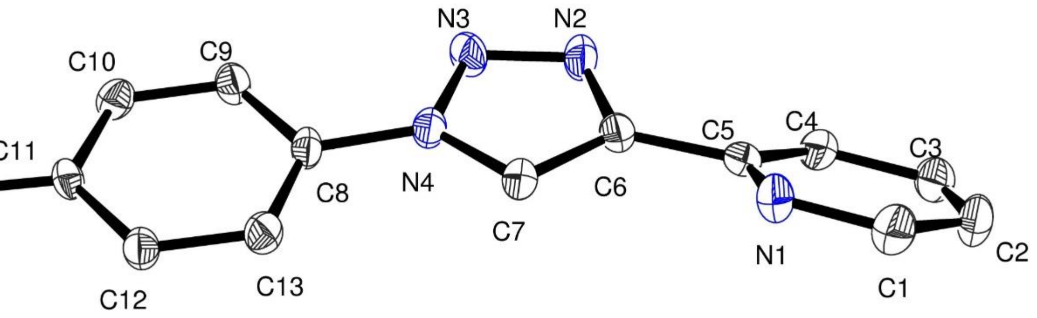

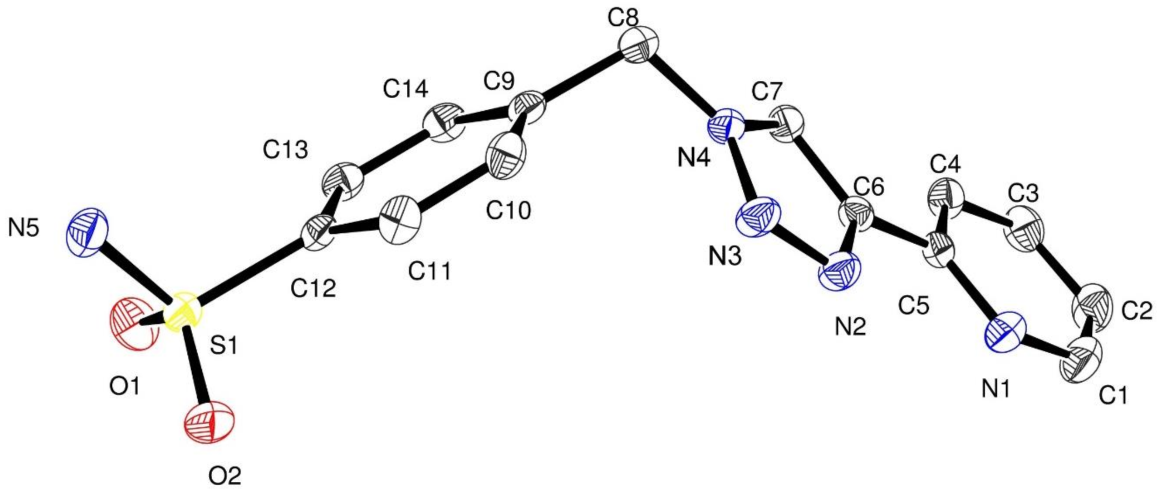


**Figure S2.** A partial view of the crystal packing of **4a** and **4b** with hydrogen bonds and π–π contacts shown as dashed lines. The purple spheres represent the centroids of the rings involved in π–π interactions.

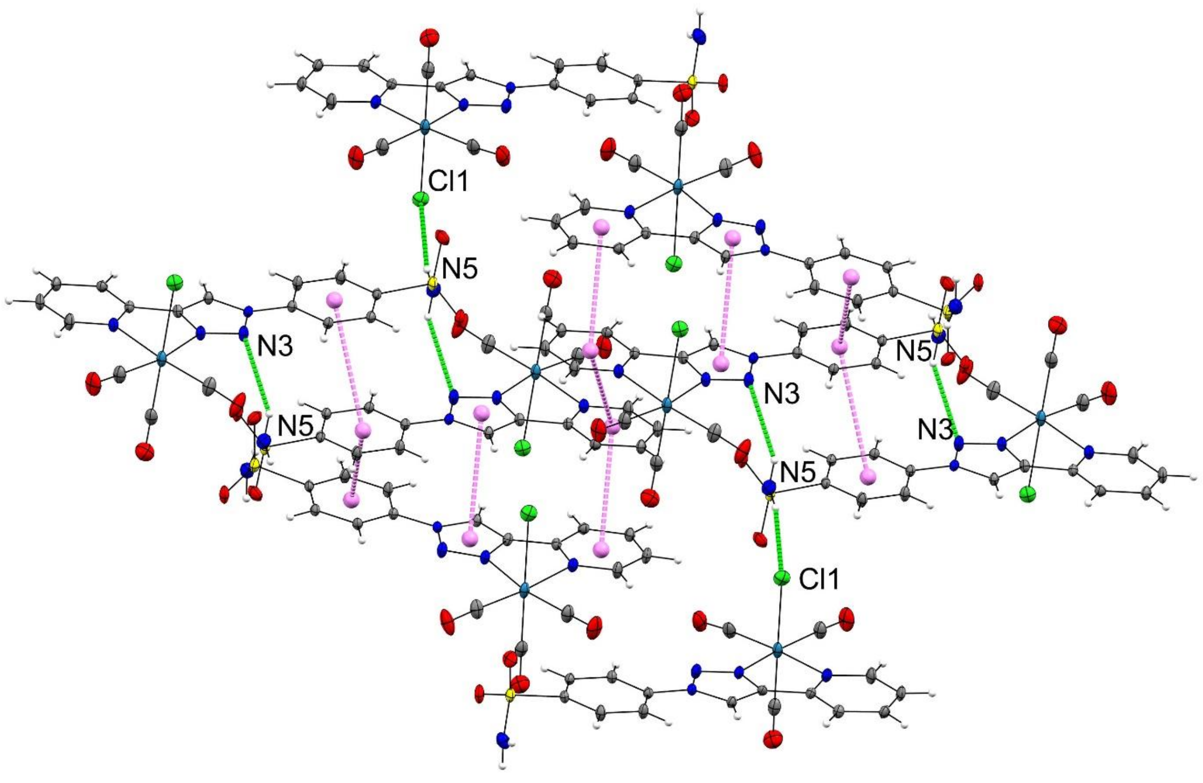

**Complex 4a**

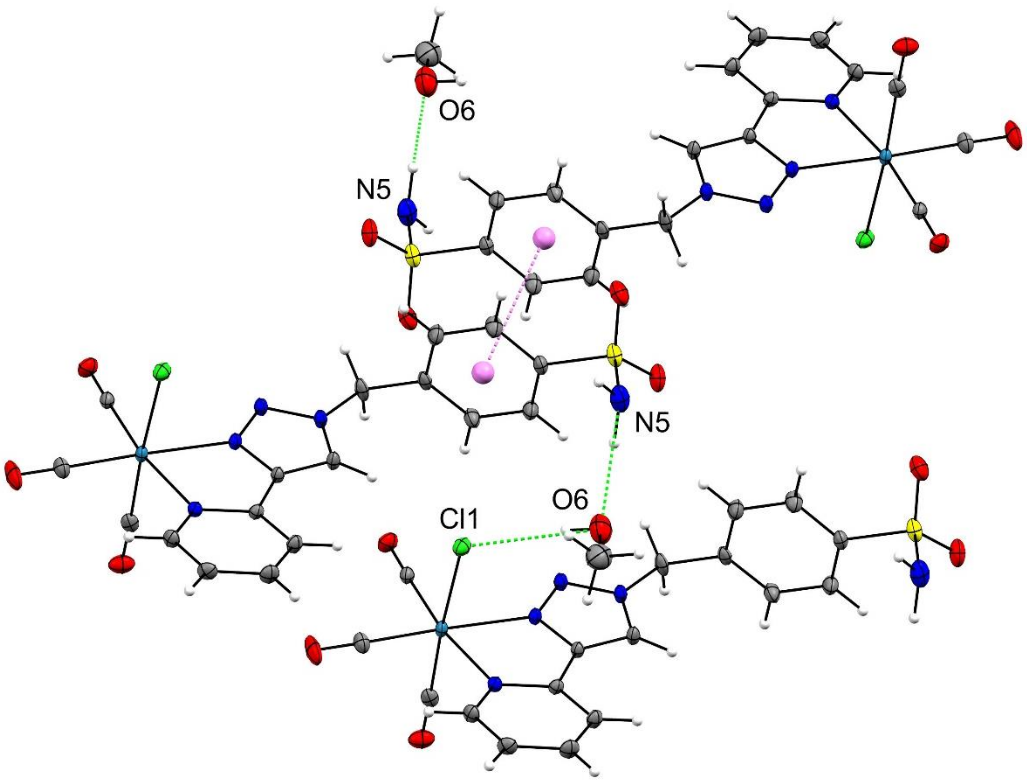


**Complex 4b**

**Figure S3.** Experimental (top) and electronic transitions calculated with TDDFT/B3LYP method (down) for **4a** and **4b**

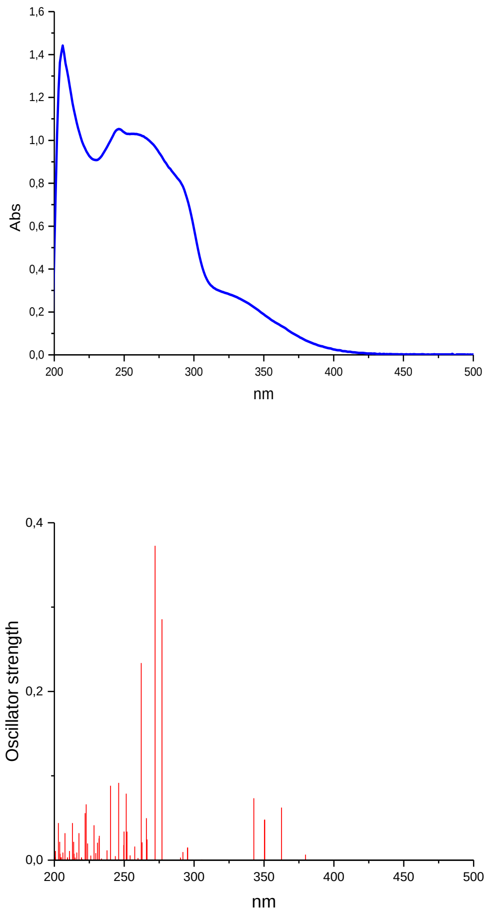
1,6
1,4
1,2
1,0
0,8
0,6
0,4
0,2
0,0
Abs
200
250
300
350
400
450
500
nm
0,4
0,2
0,0
Oscillator strength
200
250
300
350
400
450
500
nm

**4a**

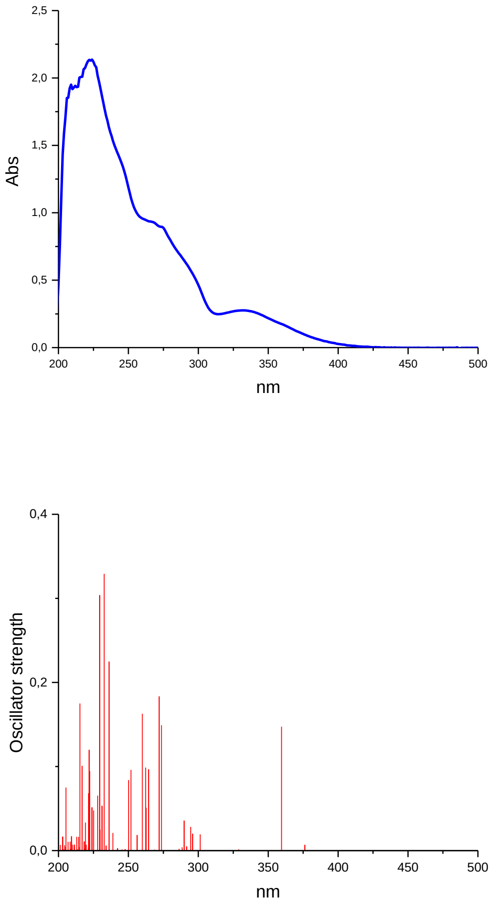
2,5
2,0
1,5
1,0
0,5
0,0
Abs
200
250
300
350
400
450
500
nm
0,4
0,2
0,0
Oscillator strength
200
250
300
350
400
450
500
nm

**4b**
